# Stress-Reducing Function of Matcha Green Tea in Animal Experiments and Clinical Trials

**DOI:** 10.3390/nu10101468

**Published:** 2018-10-10

**Authors:** Keiko Unno, Daisuke Furushima, Shingo Hamamoto, Kazuaki Iguchi, Hiroshi Yamada, Akio Morita, Hideki Horie, Yoriyuki Nakamura

**Affiliations:** 1 Department of Neurophysiology, School of Pharmaceutical Sciences, University of Shizuoka, Shizuoka 422-8526, Japan; iguchi@u-shizuoka-ken.ac.jp; 2 Tea Science Center, Graduate School of Integrated Pharmaceutical and Nutritional Sciences, University of Shizuoka, Shizuoka 422-8526, Japan; yori.naka222@u-shizuoka-ken.ac.jp; 3 Department of Drug Evaluation & Informatics, School of Pharmaceutical Sciences, University of Shizuoka, Shizuoka 422-8526, Japan; dfuru@u-shizuoka-ken.ac.jp (D.F.); m14093@u-shizuoka-ken.ac.jp (S.H.); hyamada@u-shizuoka-ken.ac.jp (H.Y.); 4 Department of Functional Plant Physiology, Faculty of Agriculture, Shizuoka University, Shizuoka 422-8529, Japan; morita.akio@shizuoka.ac.jp; 5 Institute of Fruit Tree and Tea Science, National Agriculture and Food Research Organization, Shimada 428-8501, Japan; horie@affrc.go.jp

**Keywords:** adrenal hypertrophy, anxiety, caffeine, catechin, green tea, matcha, salivary α-amylase activity, stress-reduction, theanine

## Abstract

Theanine, a major amino acid in green tea, exhibits a stress-reducing effect in mice and humans. Matcha, which is essentially theanine-rich powdered green tea, is abundant in caffeine. Caffeine has a strong antagonistic effect against theanine. The stress-reducing effect of matcha was examined with an animal experiment and a clinical trial. The stress-reducing effect of matcha marketed in Japan and abroad was assessed based on its composition. The stress-reducing effect of matcha in mice was evaluated as suppressed adrenal hypertrophy using territorially-based loaded stress. High contents of theanine and arginine in matcha exhibited a high stress-reducing effect. However, an effective stress-reducing outcome was only possible when the molar ratio of caffeine and epigallocatechin gallate (EGCG) to theanine and arginine was less than two. Participants (*n* = 39) consumed test-matcha, which was expected to have a stress-reducing effect, or placebo-matcha, where no effect was expected. Anxiety, a reaction to stress, was significantly lower in the test-matcha group than in the placebo group. To predict mental function of each matcha, both the quantity of theanine and the ratios of caffeine, EGCG, and arginine against theanine need to be verified.

## 1. Introduction

Theanine is an L-glutamate analogue and a non-protein amino acid that is particular to the tea plant (*Camellia sinensis* (L.) Kuntze) [1]. The amount of theanine, which is the most abundant amino acid in green tea leaves, depends on nitrogen supply absorbed from the roots [2]. Matcha is a fine-powdered green tea that is prepared from tea leaves protected from sunlight. When tea leaves are protected from direct sunlight, their amino acid content, especially theanine, remains high because the hydration of theanine used in the biosynthesis of catechin is lowered [3,4]. To make matcha, cultivation under shade for about three weeks is necessary before harvest [5]. Given their protection from sunlight, catechin content is lower in matcha than in other popular green teas prepared from leaves grown in sunlight [3,6]. In addition, matcha has a high content of caffeine because the buds and young leaves of *Camellia* plants contain more caffeine than mature leaves [7]. The balance of these components, such as theanine, caffeine, and catechin, determines the quality of the green tea. A higher content of amino acids indicates a higher level of “umami” ingredients. Therefore, matcha is essentially the best-grade green tea, rich in theanine and caffeine, but with a low content of catechin compared with popular green tea.

Chronic psychosocial stress is associated with the development of depression, mood disorders, and various other stress-related diseases [8,9,10]. Addressing stress-induced alterations with dietary supplements is a potential therapeutic strategy for a healthy life. Green tea is the most popular drink in Asian countries and its consumption and that of theanine has revealed health benefits and medicinal potential for several ailments [11]. Theanine exhibits an excellent stress-reducing effect on mice and humans [12,13]. However, the effect of theanine is antagonized by caffeine and epigallocatechin gallate (EGCG), which are two major components of green tea [14,15,16]. In contrast, the stress-reducing effect of theanine is enhanced by arginine (Arg), which is the second most abundant amino acid in Japanese green tea [17]. Glutamate (Glu), the third most abundant amino acid in green tea, has no effect on stress in mice [17]. To enhance the stress-reducing effect of green tea, low-caffeine green tea with reduced caffeine content can be prepared from tea leaves by irrigating them with hot water at 95 °C for three minutes. Published data showed that a significant stress-reducing effect of low-caffeine green tea was observed in participants in their 20 s, 40 s–50 s, and 80 s–90 s relative to barley tea or standard green tea [18,19,20].

Matcha is expected to have a stress-reducing effect due to its high theanine content, although this has not been scientifically proven. Previous studies described above suggested that differences in the quantities and ratios of green tea components affect the efficiency of its stress-reducing action. Therefore, the stress-reducing effect of matcha, which contains catechins, caffeine, and amino acids whose contents were measured, was evaluated in an animal (mouse) experiment. Mice were stressed using territorial conflict between male mice [14]. The stress-reducing effect of matcha was evaluated as the suppression of adrenal hypertrophy in stressed mice because adrenal glands are sensitive to stress [14]. The relationship between suppressed adrenal hypertrophy and quantities or ratios of matcha tea components was examined. Based on these data, test-matcha with contents expected to have a stress-reducing effect, as well as placebo-matcha with contents expected to have no stress-reducing effect, were selected for a clinical trial. Participants, who were selected for a double-blind randomized controlled trial, consumed matcha (3 g) suspended in 500 mL water daily. They were fifth year college students of the University of Shizuoka, School of Pharmaceutical Sciences, Japan, who were assigned to a pharmacy practice outside the university, such as a hospital or a pharmacy. Commitment to a new environment provides a stressful condition for young students. Since anxiety is a reaction to stress, to assess the anxiety of participants, the state-trait anxiety inventory (STAI) test was administered before and on the eighth day of pharmacy practice. In addition, to assess the physiological stress response, the activity of salivary α-amylase activity (sAA), an oral cavity enzyme, was measured as a stress marker of sympathetic excitement [15]. This enzyme rapidly increases in response to physiological and psychosocial stress [21]. We examined whether test-matcha was able to reduce participants’ stress. Finally, based on the quantity and ratio of components of each matcha, 76 matcha samples sold in Japan and 67 samples sold abroad were evaluated to determine whether mental function such as anxiety and stress could be expected when humans consume these forms of matcha.

## 2. Materials and Methods

### 2.1. Measurement of Tea Components by High-Performance Liquid Chromatography

The components in matcha were measured by high performance liquid chromatography (HPLC) as described previously [17]. We focused on the contents of theanine, arginine, caffeine, and EGCG. In brief, according to the method of Horie et al. [5], catechins and caffeine were measured by HPLC at 280 nm (SCL-10Avp, Shimadzu, Kyoto, Japan; Develosil packed column ODS-HG-5, 150 × 4.6 mm, Nomura Chemical Co. Ltd., Seto, Japan). Free amino acids (Arg, alanine, aspartic acid (Asp), asparagine (Asn), glutamic acid (Glu), glutamine (Gln), *γ*-amino butyric acid (GABA), serine (Ser), and theanine) were measured by HPLC as described above using glycylglycine as the internal standard [22]. These amino acids were detected at an excitation wavelength of 340 nm and at an emission wavelength of 450 nm (RF-535 UV detector, Shimadzu, Kyoto, Japan). Based on these data, seven matcha samples were selected for the animal experiment.

### 2.2. Animal Studies

#### 2.2.1. Animals and Stress Experiment

Male ddY mice (Slc: ddY, four weeks old) were purchased from Japan SLC Co. Ltd. (Shizuoka, Japan) and kept under conventional conditions in a temperature- and humidity-controlled environment with a 12/12 h light/dark cycle (light period, 8:00 a.m.–8:00 p.m.; temperature, 23 ± 1 °C; relative humidity, 55 ± 5%). Four-week-old mice were reared in a group of six in a cage for five days to allow them to adapt to co-habitation. Mice were fed a normal diet (CE-2; Clea Co. Ltd., Tokyo, Japan) and water *ad libitum*. All experimental protocols were approved by the University of Shizuoka Laboratory Animal Care Advisory Committee (approval no. 166197) and were in accordance with the guidelines of the U.S. National Institute of Health for the Care and Use of Laboratory Animals. To apply psychosocial stress to mice, confrontational rearing was performed in a standard polycarbonate cage that was divided into two identical subunits by a stainless-steel partition as previously described [14]. In brief, two mice were reared in a partitioned cage for one week (single rearing) to establish territorial consciousness. Then, the partition was removed to expose the mice to confrontational stress for 24 h (confrontational rearing). Adrenal glands, a critical stress-responsible organ, became significantly enlarged after confrontational rearing and peak size was reached at 24 h. The phenomenon continues for at least one week [14]. In mice under confrontational rearing, adrenal-hypertrophy, change in diurnal rhythm of corticosterone, and depression-like behavior have been observed. In addition, adrenal hypertrophy was suppressed by the intake of the antidepressant and anxiolytic drug, diazepam [14]. Since the suppression of adrenal hypertrophy is thought to be related to antidepressant and anxiolytic effects, the stress-reducing and anxiolytic effects of matcha in mice was evaluated by weighing the adrenal glands. Each cage was placed in a styrofoam box (width 30 cm, length 40 cm, height 15 cm) to avoid visual social contact between cages.

#### 2.2.2. Ingestion of Matcha or Tea Components by Mice

**Scheme 1:**
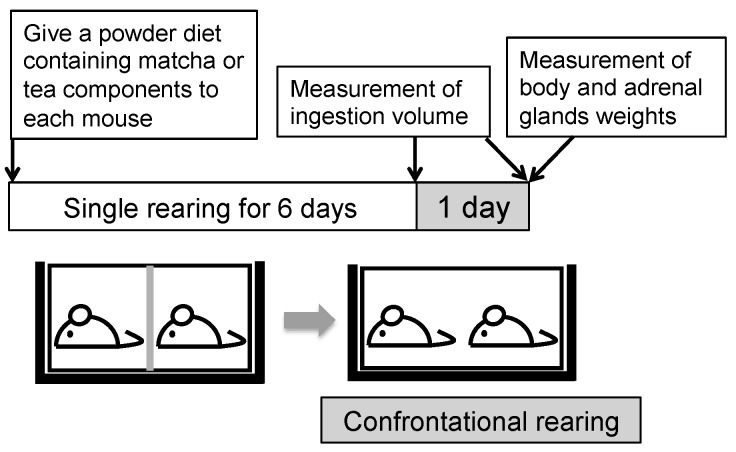
Study design of the animal experiment.

The effect of matcha was examined in 15 groups of mice (4–8 mice/group, *n* = 86). Mice consumed matcha or tea components in a powder diet of CE-2 ad libitum for seven days (single rearing for six days and confrontational rearing for one day) (Scheme 1). The experimental dose was set based on the consumption in humans. Since about 2–3 g of matcha is used per cup in the tea ceremony in Japan, the intake (2–3 g/60 kg body weight) in humans corresponds to 33–50 mg/kg in mice. Based on this and considering the difference between humans and mice, the effect of matcha was examined at 0, 10, 17, 33, 50, and 100 mg/kg (body weight) doses in mice. Ingested food weight was measured using a special bait box for measurement of exact ingestion volume (Roden CAFE^®^, Oriental Yeast Co., Ltd., Tokyo, Japan). Mouse body weight was measured on the last day of the experiment. Seven matcha samples were used for the experiment. The stress-reducing effect was compared among matcha samples no. 1–5, at a dose of 33 mg/kg, and no. 6–7, at 50 mg/kg. Tea components used were as follows: L-theanine (Suntheanine; Taiyo Kagaku Co. Ltd., Yokkaichi, Japan), EGCG (Sunphenon EGCg, Taiyo Kagaku Co. Ltd., Yokkaichi, Japan), caffeine, and Arg (Wako Pure Chemical Co. Ltd., Osaka, Japan). The interaction of these tea components was examined on adrenal hypertrophy in stressed mice. The stress-reducing effect of tea components was compared among four groups of mice as follows: group 1 was control mice fed a powder diet; group 2 included mice fed a diet containing only theanine; group 3 included mice fed a diet containing theanine, caffeine, and EGCG; and group 4 included mice fed a diet containing theanine, caffeine, EGCG, and Arg. The concentration of theanine was 0.32 mg/kg. The molar ratio of each component was that of matcha sample no. 6, i.e., the molar ratio of theanine:caffeine:EGCG:Arg was 1:2:1:0.7. At the end of the 24 h of confrontational rearing, mice were sacrificed and adrenal glands were weighed.

### 2.3. Human Studies

#### 2.3.1. Participants

Thirty-nine healthy students (23 ± 1.1 years old, 23 men and 16 women) who participated in the experiment received verbal and written information about the study. They signed an informed consent form before participating in the study. None of the participants indicated any acute or chronic diseases, regular intake of medication, or habitual smoking. Participants were instructed to drink mainly the test-matcha tea, and to not consume theanine- and caffeine-rich beverages, such as other teas, including green tea, coffee, black tea, and soda, throughout the experiment. They were also instructed not to consume caffeine-rich foods such as chocolate and candies. Participants were allowed to drink water freely, but were not permitted to consume alcohol at night to eliminate psychological effects due to alcohol intake. They were randomly divided into two groups: test-matcha (*n* = 19) and placebo-matcha (*n* = 20).

The participants were assigned to a practice outside the university, either in a hospital or at a pharmacy, for 11 weeks. Seven days of routine university life and the first eight days of the students’ practice program were analyzed. The study was conducted in accordance with the Declaration of Helsinki and Ethical Guidelines for Medical and Health Research Involving Human Subjects (Public Notice of the Ministry of Education, Culture, Sports, Science, and Technology and the Ministry of Health, Labour and Welfare, 2008). This study was approved in Japan, and the study protocol, which was approved by the Ethics Committee of the University of Shizuoka (no. 28-60), was registered at the University Hospital Medical Information Network (UMIN) (registration no. UMIN26905). The study period was from April to May 2017.

#### 2.3.2. Procedure

This study was based on a group comparison design and participants were randomly assigned to test- or placebo-matcha groups. The test- and placebo-matcha were packed 3 g each into exactly same-shaped bags. The participants did not know whether they were consuming test- or placebo-matcha. The intake of test- or placebo-matcha tea started from seven days prior to pharmacy practice and continued for eight days into the practice period, for a total of 15 days (Table 1). Since anxiety is a reaction to stress, to assess the anxiety of participants, the state-trait anxiety inventory (STAI) test (Japanese STAI Form X-1, Sankyobo, Kyoto, Japan) was administered before pharmacy practice and on the eighth day of pharmacy practice. In this study, the state-anxiety of participants was compared between test- and placebo-groups.

A questionnaire that included feedback on their physical condition, subjective stress, and achievement emotion was assigned for 15 days after each day’s practice. The physical condition of participants was assigned an ordinal scale (5, very good; 4, good; 3, normal; 2, slightly bad; 1, bad). Subjective stress was evaluated using visual analogue scales (VAS: 0–10) from very relaxed to highly stressed. Achievement emotion was assigned an ordinal scale (5, completely; 4, better; 3, a little better; 2, a little worse; 1, much worse). Sleeping hours were also recorded. Each participant’s median data for the last three days at the university and pharmacy practice were used for statistical analysis considering the influence of consecutive ingestion of matcha.

#### 2.3.3. Measurement of sAA

To assess the physiological stress response, sAA was measured using a colorimetric system (Nipro Co., Osaka, Japan) [23]. In brief, salivary amylase hydrolyzes a substrate, 2-chloro-4-nitrophenyl-4-*O*-β-d-galactopyranosylmaltoside, in the presence of maltose, a competitive inhibitor. The color of a reagent strip turns from white to yellow in this reaction, and changes are quantified using a salivary amylase monitor. One unit of activity (U) per mass of enzyme is defined as the production of 1 µmol of the reduction sugar, maltose, in 1 min (NC-IUBMB, EC 3.2.1.1). Prior to sampling, participants washed their mouths with water. After saliva was collected for 30 s using a sampling tip, participants measured their own sAA immediately. Saliva was measured in the morning after waking up (sAAm). To establish a sAA baseline, participants recorded measurements every morning for three days during routine daily life at the university before intake of matcha. Next, the participants measured sAA every morning and every evening (sAAe) for seven days during routine daily life at the university, and successively for eight days during pharmacy practice (Table 1). Each participant’s median sAA of the last three days at the university and pharmacy practice was used for statistical analysis considering the influence of consecutive ingestion of matcha.

### 2.4. Statistical Analyses

Data are expressed as the mean ± standard error of the mean (SEM). Statistical analyses were performed using Student’s *t*-test and one-way analysis of variance (ANOVA) followed by Bonferroni’s post-hoc test for multiple comparisons. All statistical analyses were performed in a statistical analysis program, JMP ver.13 (SAS Institute Inc., Cary, NC, USA). Differences were considered to be significant at *p* < 0.05.

## 3. Results

### 3.1. Animal Study

#### 3.1.1. Anti-Stress Effects of Matcha in a Mouse Model of Psychosocial Stress

**Figure 1:**
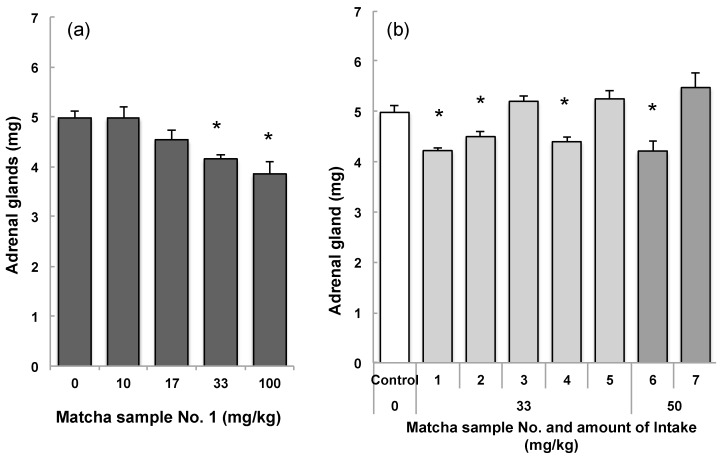
Suppression of adrenal hypertrophy by matcha intake in stressed mice. (**a**) Mice consumed a matcha sample (no. 1) in a powder diet for seven days (single rearing for six days and confrontational rearing for one day). Since each mouse (ca. 30 g) ingested about five grams of diet per day, the intake of matcha was set to 0, 10, 17, 33, and 100 mg/kg; (**b**) Mice were fed a diet containing matcha from no. 1 to no. 5 at a concentration of 33 mg/kg, and from no. 6 to no. 7 at a concentration of 50 mg/kg. Each bar shows the mean ± SEM (*n* = 4–8; * *p* < 0.05).

The relationship between the amount of matcha intake and suppression of adrenal hypertrophy was examined using matcha sample no. 1, whose theanine content represented the median of seven samples. The difference in matcha concentration did not affect food intake or body weight. The weight of adrenal glands increased to 5.0 ± 0.2 mg in mice after confrontational rearing from about 4.0 mg before confrontation. The results show that adrenal hypertrophy was significantly suppressed in mice that ingested more than 33 mg/kg of matcha (Figure 1a).

The stress-reducing effect was then compared among the seven samples of matcha. Adrenal hypertrophy was significantly suppressed in mice that consumed matcha sample nos. 1, 2, 4, and 6. Suppression of stress was not observed in mice that consumed matcha samples nos. 3, 5, or 7 (Figure 1b). The former samples had less total amino acid content than the latter batch of matcha samples (Table 2).

#### 3.1.2. Relationship between Tea Components in Each Matcha Sample and Suppression of Adrenal Hypertrophy

**Figure 2:**
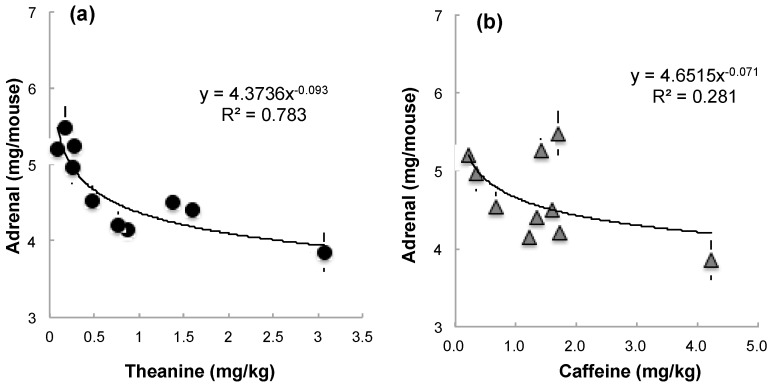
Correlations between adrenal hypertrophy and ingestion of matcha components in stressed mice. The amounts of (**a**) theanine and (**b**) caffeine were calculated from the matcha-containing feed that each mouse consumed, as shown in Table 3. Each point and bar shows the mean ± SEM (*n* = 4–8).

We confirmed that theanine and Arg have a significant stress-reducing effect [17]. These are the most and the second most abundant amino acids in Japanese matcha. However, the third and fourth most abundant amino acids, glutamate and glutamine, had no effect. Therefore, the actual amount of theanine and arginine ingested by mice from matcha was calculated (Table 3). To suppress adrenal hypertrophy in mice, theanine is needed at 0.32 mg/kg or more [17]. This explains the lack of a stress-reducing effect in mice that ingested sample nos. 3, 5, and 7 (Table 3). The relationship between theanine intake and adrenal hypertrophy showed a dose-dependent suppression of the latter (Figure 2a, *R*^2^ = 0.783). A similar correlation was observed for Arg (*R*^2^ = 0.783). There was no relationship between caffeine intake and adrenal hypertrophy (Figure 2b, *R*^2^ = 0.281).

Although the effect of theanine at 0.48 mg/kg (sample no. 1, 17 mg/kg) was slightly low, the coexistence of caffeine and EGCG may have counteracted the effect of theanine. All matcha samples contained both, equal, or higher molar amounts of caffeine and EGCG.

**Figure 3:**
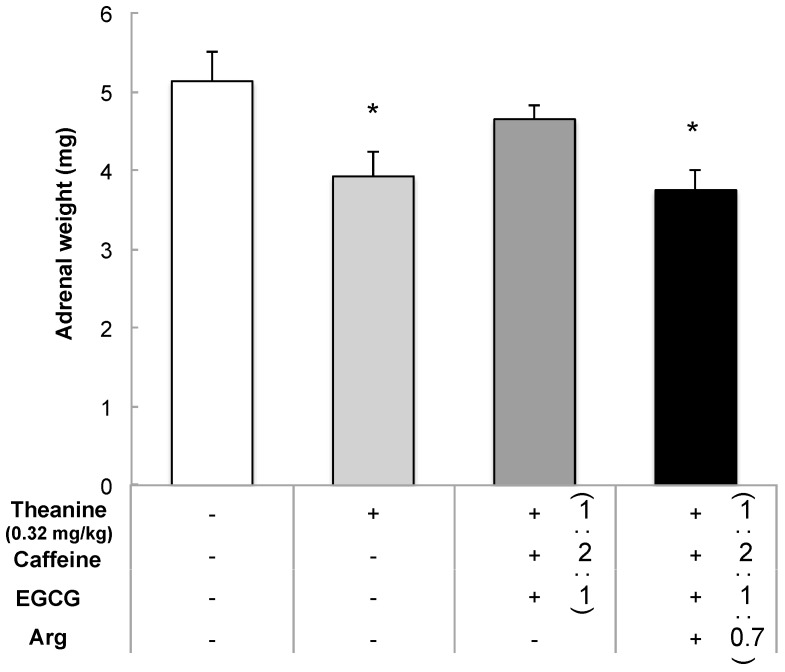
Interaction of tea components on adrenal hypertrophy in stressed mice. Mice consumed powder diet containing theanine, caffeine, EGCG, and Arg for seven days (single rearing for six days and confrontational rearing for one day). Control mice were fed a powder diet, CE-2. The concentration of theanine was 0.32 mg/kg. Mice ingested feed that contained each component as molar ratios of matcha sample no. 6, i.e., the molar ratio of theanine:caffeine:EGCG:Arg was 1:2:1:0.7. Each bar shows the mean ± SEM (*n* = 4; * *p* < 0.05).

To examine the relationship among theanine, Arg, caffeine, and EGCG, mice ingested feed that contained each component as a molar ratio of sample no. 6 (Table 4). When the concentration of theanine was 0.32 mg/kg, adrenal hypertrophy was significantly suppressed by the ingestion of theanine (Figure 3). However, the action of theanine was antagonized by the coexistence of two-fold higher molar concentration of caffeine and an equal molar ratio of EGCG (Figure 3). In contrast, the coexistence of a 0.7 molar ratio of Arg to theanine recovered the counteraction of caffeine or EGCG (Figure 3), suggesting that Arg plays an essential role in the suppression of adrenal hypertrophy. The molar ratio of caffeine and EGCG to theanine and Arg was ≤1.8 in the effective samples, but was ≥3.6 in the non-effective samples (Table 4). These results indicate that the molar ratio of Arg, caffeine, and EGCG additionally affects theanine.

### 3.2. Human Study

#### 3.2.1. Effect of Matcha Ingestion on Stress in Students Assigned Pharmacy Practice Outside the University

**Figure 4:**
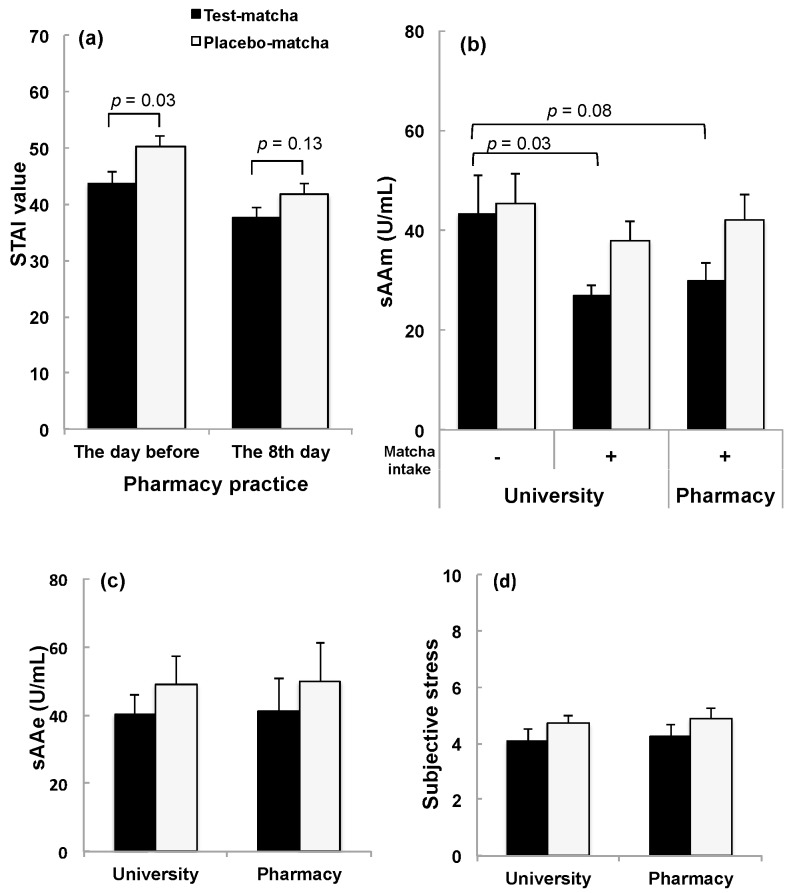
Effect of matcha ingestion on students during university and pharmacy practice. (**a**) The STAI test was administered before and on the eighth day of pharmacy practice. (**b**, left bars) The level of sAAm was measured in participants every morning for three days during routine daily life at the university before intake of matcha as the baseline. (**b**, middle and right bars) After the ingestion of matcha started, median sAAm of each participant for the last three days at the university and pharmacy practice was used for statistical analysis. The median of (**c**) sAAe and (**d**) subjective stress of each participant of the last three days at the university and pharmacy practice are shown. Each bar shows the mean ± SEM (test matcha, *n* = 19; placebo matcha, *n* = 20).

Participants consumed three grams of matcha daily that were suspended in 500 mL of room-temperature water. Test-matcha was sample No. 6 and placebo-matcha was sample no. 7. These matcha samples were selected based on the mice data shown in Table 2 and Table 4. Before pharmacy practice, the STAI value of those participants that consumed test-matcha was significantly lower than that of placebo-matcha (*p* = 0.03, Figure 4a). On the eighth day of pharmacy practice, the raw values and the difference between the means of these two groups was low (*p* = 0.13).

These data indicate that the intake of test-matcha was effective for the specific suppression of anxiety before practice outside the university. Although the basal level of sAAm before the intake of matcha was not different between the participants of the test- and placebo-matcha groups, the level at university was significantly lowered by the intake of test-matcha (*p* = 0.03, Figure 4b). Similarly, in pharmacy practice, the level of sAAm tended to be lower in the test-matcha group (*p* = 0.08, Figure 4b). In both groups, the sAA levels increased in the evening after practice (Figure 4c). The subjective stress that was recorded after practice tended to be slightly lower in the test group than in the placebo group, but it was not significantly different between both groups (Figure 4d). Nerve excitation was commonly down-regulated by the next morning [24]. Test-matcha may regulate recovery rather than suppress excitation. Differences in sex were not observed in STAI and sAA values. Other parameters such as physical condition, sleeping time, and achievement emotion were not significantly different between the two groups during pharmacy practice (Table 5).

#### 3.2.2. Composition of Matcha Marketed in Japan and Overseas

**Figure 5:**
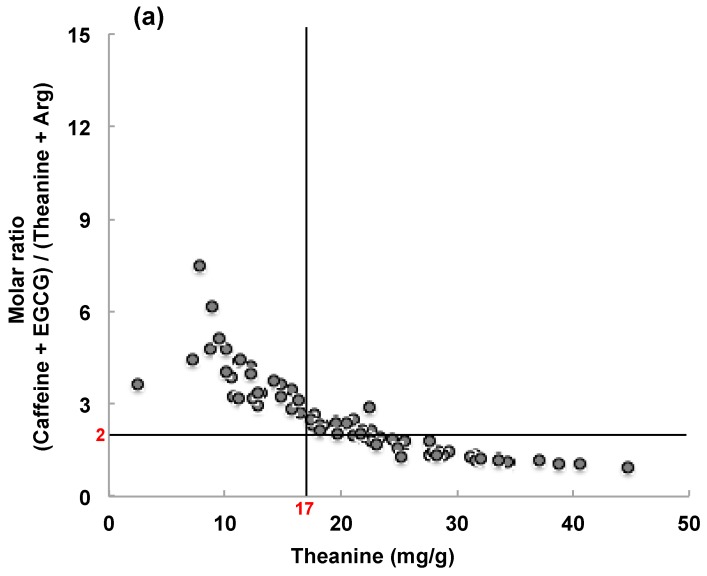

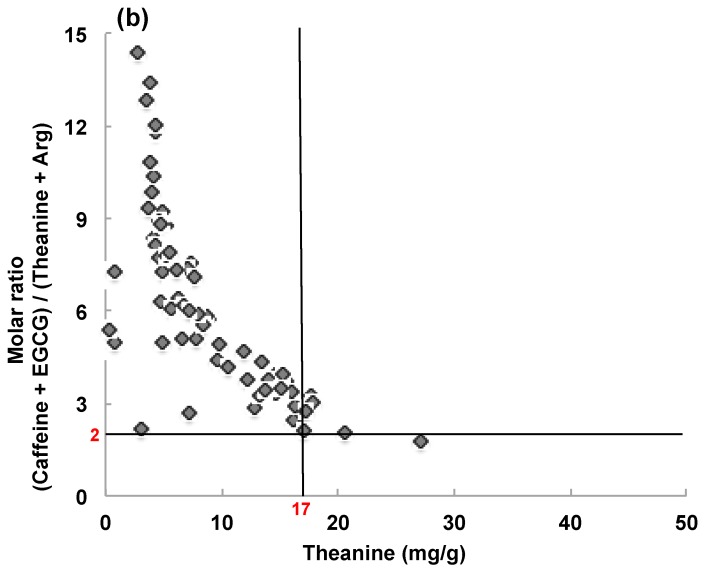
The amount of theanine and the molar ratio of caffeine and EGCG to theanine and Arg in (**a**) 76 samples of matcha marketed in Japan and (**b**) 67 samples of matcha marketed overseas. Based on data in humans, samples in the section, theanine > 17 mg/g, and molar ratio of caffeine and EGCG to theanine and Arg < 2 are expected to show a stress-reducing effect.

The components of matcha marketed in Japan (76 samples) and overseas (67 samples) were measured (supplemental data; Tables S1 and S2). When a human takes three grams of matcha in a day, a clinical experiment showed that matcha samples that contain levels of theanine exceeding 17 mg/g might have a stress-reducing effect. As a result, 50 out of 76 samples in Japan met this condition. In contrast, of the 67 samples sold overseas, six samples met this condition (Figure 5a,b). In addition to this, the molar ratio of caffeine and EGCG to theanine and Arg might need to be lower than two. The result indicated that 32 out of 76 samples in Japan met this condition (Figure 5a). Only one sample sold overseas met the condition (Figure 5b).

## 4. Discussion

Based on the quantities and ratios of matcha components, the stress-reducing effect of matcha was evaluated by mice experiments using seven kinds of matcha. These matcha samples were selected based on differences in theanine content. Since the amount of theanine in the tea leaves depends on the amount of nitrogen supply in the soil, one matcha with low theanine content was sourced from organic cultivation. The matcha sample containing high amounts of EGCG might be prepared from leaves that were not fully protected from the sunlight before harvest. Additionally, a sample with low amounts of all components was a matcha product containing additives.

Since theanine and Arg reduce stress and caffeine and EGCG antagonizes the action of theanine [16,17], we examined how these levels affect adrenal hypertrophy in stressed mice. In mice, adrenal hypertrophy was suppressed when the amount of theanine ingested was 0.32 mg/kg or more. Since the effect of theanine is cancelled by caffeine and EGCG, a molar ratio of caffeine and EGCG to theanine and Arg must be less than 3.6. Although EGC has a stress-reducing effect, EGC needed to be more than 10 times higher than EGCG [17]. As EGC was not higher than EGCG in matcha, the effect of EGC was not considered. Matcha essentially contains a high amount of caffeine, but if theanine is sufficiently high, the anti-theanine action of caffeine is counteracted. Catechin in matcha is less than in general green teas, but its increase is accompanied by a decrease in theanine. Therefore, an increase in catechin in matcha potentially increases the reduction of the stress-reducing effect of matcha.

Using test-matcha, which meets these conditions, we conducted a study in humans. Whereas the distribution of sAAm, a marker of physiological stress, was similar in the test and placebo groups before matcha was consumed, anxiety (STAI) and physiological stress (sAAm) decreased when test-matcha was consumed. The significant decrease in sAAm at university due to the intake of test-matcha may have contributed to the decrease in anxiety before pharmacy practice. Although caffeine consumption is reportedly linked to anxiety sensitivity [25,26], the caffeine content in test- and placebo-matcha were not different (Table 2). Additionally, the amount of caffeine ingested by participants was about 40 mg/day, which is about one-quarter cup of coffee, so drinking it every day would not cause a problem. Rather, in the case of matcha, the counteracting effects of caffeine and EGCG against theanine and Arg are considered to be important.

Whereas EGCG has been reported to have anxiolytic effects [27], the antagonistic effect of EGCG on theanine may be due to an increased excitatory synaptic connection by EGCG [28]. Glutamate is the main excitatory neurotransmitter and EGCG reportedly facilitates the release of glutamate [29]. Although central glutamatergic activity is crucial to cognitive function, excessive release of glutamate causes excessive excitation. The level of GABA, the main inhibitory neurotransmitter in the brain, is increased by theanine ingestion [30,31]. These data suggest that different ratios of EGCG/theanine cause a different balance of excitatory and inhibitory neurotransmitters such as glutamate and GABA.

Since the active component of matcha could be applied to evaluate the stress-reducing effect of matcha in humans, we then tried to assess the stress-reducing effect of matcha marketed in Japan and overseas. When people ingested three grams of these matcha per day, we examined whether stress-reducing effects would be expected. The results showed that, about 42% of matcha sold in Japan was expected to suppress stress versus only one of the matcha marketed overseas, because the amounts of theanine and Arg were low, and the amounts of caffeine and EGCG were high. Matcha is essentially rich in theanine and low in catechin, but not all marketed matcha satisfied this condition. This indicates that greater attention is needed to evaluate the mental function of matcha. Although the number of people drinking green tea as a health beverage is increasing in many countries, low-grade green teas with low amino acid contents are also sold as “matcha” [32]. Many matcha marketed overseas had low amino acids contents (Figure 5, Table S2). The counteracting effect of caffeine and EGCG on theanine is hardly considered. This may cause confusing results. For example, the effect of matcha tea on mood and cognitive performance has been examined [33]. The mood state measured by a Profile of Mood State (POMS) was not significantly changed by the ingestion of four grams of matcha tea. The matcha tea that those authors used contained 67 mg theanine, 280 mg EGCG, and 136 mg caffeine. Theanine content was slightly less than 17 mg/g. Although the content of Arg was not reported, the molar ratio of caffeine and EGCG against theanine was higher than two. This suggests that the result of POMS might have been different if those authors had used another matcha sample with a lower content of caffeine and EGCG. EGCG has been reported to have various beneficial functions [34]. However, when expecting mentally positive functions of matcha in anxiety, stress, and mood, the quantities of theanine and Arg must be high, and the ratios of caffeine and EGCG against theanine must be low.

## 5. Conclusions

We evaluated the effects of quantity and ratio of matcha components on its stress-reducing properties using an animal model of psychosocial stress. The stress-reducing effect in humans was confirmed using two kinds of matcha selected based on the animal study. In addition, we assessed the stress-reducing effect of matcha marketed in Japan and overseas. As a result, 42% of matcha samples marketed in Japan, and only one sample marketed abroad, were expected to have a stress-reducing effect. When using matcha samples to study mental function, a quality check is critical.

## Figures and Tables

**Table 1:** Experimental procedure.

Practice	University				Pharmacy	
Days	3	3–10	7		8	
Matcha intake	(−)	(−)	+		+	
sAAm	+	(−)	+		+	
STAI	(−)	(−)	(−)	+	(−)	+

sAAm: salivary α-amylase activity in the morning, STAI: state-trait anxiety inventory. That was administered before pharmacy practice and on the eighth day of pharmacy practice.

**Table 2:** Ingredient composition in each matcha sample.

**Sample**	**Amino Acids (mg/g)**								
**No**	**Theanine**	**Arg**	**Glu**	**Asp**	**Asn**	**Ser**	**Gln**	**GABA**	**Total**
**1**	28.99 ± 0.10	12.50 ± 0.04	7.52 ± 0.02	6.95 ± 0.02	2.90 ± 0.01	1.25 ± 0.00	1.08 ± 0.01	0.25 ± 0.00	61.45 ± 0.19
**2**	38.77 ± 0.16	18.19 ± 0.02	7.58 ± 0.05	8.47 ± 0.05	3.36 ± 0.01	1.84 ± 0.01	1.34 ± 0.01	0.22 ± 0.00	79.77 ± 0.43
**3**	2.48 ± 0.03	0.36 ± 0.00	0.48 ± 0.00	0.65 ± 0.01	0.09 ± 0.00	0.13 ± 0.00	0.11 ± 0.00	0.05 ± 0.00	4.35 ± 0.04
**4**	44.65 ± 1.73	20.39 ± 1.06	5.86 ± 0.16	7.95 ± 0.19	4.01 ± 0.12	1.74 ± 0.05	3.20 ± 0.11	0.23 ± 0.01	88.02 ± 3.41
**5**	7.87 ± 0.17	1.41 ± 0.06	2.64 ± 0.03	2.80 ± 0.02	0.59 ± 0.02	0.57 ± 0.03	0.51 ± 0.01	0.14 ± 0.00	16.52 ± 0.26
**6**	17.41 ± 0.18	12.33 ± 0.32	5.37 ± 0.04	7.60 ± 0.14	3.18 ± 0.03	1.34 ± 0.01	0.85 ± 0.01	0.13 ± 0.00	48.21 ± 0.70
**7**	3.91 ± 0.09	1.43 ± 0.11	2.82 ± 0.05	2.94 ± 0.06	0.82 ± 0.02	0.58 ± 0.01	0.38 ± 0.01	0.26 ± 0.01	13.15 ± 0.37
**Sample**	**Caffeine**	**Catechin (mg/g)**							
**No**	**(mg/g)**	**EGCG**	**ECG**	**EGC**	**EC**				
**1**	39.95 ± 0.19	59.34 ± 0.41	12.44 ± 0.07	14.86 ± 0.06	4.26 ± 0.01				
**2**	44.43 ± 0.47	57.20 ± 0.84	13.81 ± 0.21	9.68 ± 0.04	3.44 ± 0.02				
**3**	5.95 ± 0.02	13.15 ± 0.08	2.20 ± 0.01	4.09 ± 0.02	0.87 ± 0.00				
**4**	37.19 ± 0.36	48.44 ± 0.53	9.96 ± 0.15	8.40 ± 0.06	2.50 ± 0.04				
**5**	40.35 ± 0.70	86.76 ± 1.47	06.09 ± 0.34	28.76 ± 0.38	6.48 ± 0.13				
**6**	38.95 ± 0.55	49.15 ± 0.69	25.41 ± 0.69	9.91 ± 0.09	3.29 ± 0.03				
**7**	37.06 ± 0.23	64.61 ± 4.30	30.54 ± 1.51	31.21 ± 0.40	6.75 ± 0.11				

Data represents mean ± SEM (*n* = 2–3). ECG, epicatechin gallate; EGC, epigallocatechin; EC, epicatechin.

**Table 3:** Adrenal weight in mice that ingested each matcha sample, and the actual amount of each component that was ingested by mice.

Matcha	Concentration	Adrenal	Matcha Intake	Theanine	Arginine	Caffeine	EGCG
No.	(mg/kg)	(mg/Mouse)	(mg/kg)	(mg/kg)	(mg/kg)	(mg/kg)	(mg/kg)
1	10	4.97 ± 0.23	8.7 ± 2.2	0.25 ± 0.03	0.11 ± 0.01	0.35 ± 0.04	0.51 ± 0.05
1	17	4.53 ± 0.19	16.7 ± 0.4	0.48 ± 0.01	0.21 ± 0.01	0.67 ± 0.01	0.99 ± 0.02
1	33	4.15 ± 0.09	30.2 ± 1.5	0.88 ± 0.04	0.38 ± 0.02	1.21 ± 0.06	1.79 ± 0.09
1	100	3.85 ± 0.25	105.9 ± 5.0	3.07 ± 0.14	1.32 ± 0.06	4.23 ± 0.20	6.28 ± 0.30
2	33	4.50 ± 0.09	35.5 ± 0.2	1.38 ± 0.01	0.65 ± 0.00	1.58 ± 0.01	2.03 ± 0.01
3	33	5.20 ± 0.10	35.6 ± 1.1	0.09 ± 0.00	0.01 ± 0.00	0.21 ± 0.01	0.47 ± 0.01
4	33	4.40 ± 0.09	35.8 ± 0.3	1.60 ± 0.01	0.73 ± 0.01	1.33 ± 0.01	1.73 ± 0.01
5	33	5.25 ± 0.17	34.8 ± 0.6	0.27 ± 0.01	0.05 ± 0.00	1.40 ± 0.02	3.02 ± 0.05
6	50	4.21 ± 0.19	44.3 ± 1.9	0.77 ± 0.03	0.55 ± 0.02	1.73 ± 0.07	1.79 ± 0.09
7	50	5.48 ± 0.29	45.7 ± 1.7	0.18 ± 0.01	0.07 ± 0.00	1.69 ± 0.06	2.95 ± 0.11

Data represents mean ± SEM (*n* = 4–8).

**Table 4:** Molar ratios in matcha samples.

Matcha No.	Caffeine/Theanine	EGCG/Theanine	Arg/Theanine	(Caffeine + EGCG)/(Theanine + Arg)
1	1.23	0.78	0.43	1.41
2	1.03	0.56	0.47	1.08
3	2.17	2.01	0.15	3.63
4	0.75	0.41	0.46	0.79
5	4.60	4.19	0.18	7.45
6	2.01	1.07	0.71	1.80
7	8.50	6.28	0.37	10.79

**Table 5:** Effect of consumption of test- and placebo-matcha by students at university and pharmacy practice on psychosocial responses, as assess from a questionnaire.

Questionnaire Item	University			Pharmacy		
	Matcha		*p*	Matcha		*p*
	Test	Placebo	value	Test	Placebo	value
Physical condition (1−5)	3.55 ± 0.16	3.32 ± 0.13	0.30	3.44 ± 0.12	3.36 ± 0.12	0.62
Sleep time (h)	6.33 ± 0.15	6.20 ± 0.18	0.71	6.15 ± 0.17	6.40 ± 0.19	0.40
Achievement emotion (1−5)	(−)	(−)		3.49 ± 0.20	3.49 ± 0.16	0.82

## Supplementary Materials

The following are available online at http://www.mdpi.com/2072-6643/10/10/1468/s1, Table S1: Components of matcha marketed in Japan (mg/g), Table S2: Components of matcha marketed abroad (mg/g).
