# Supplementary material for: Stress-Reducing Function of Matcha Green Tea in Animal Experiments and Clinical Trials
Source: Nutrients. 2018 Oct 10;10(10):1468. doi: 10.3390/nu10101468 (PMC6213777; doi:10.3390/nu10101468)
Supplement: Supplementary file 1 [file nutrients-10-01468-s001.pdf]

Table s1. Components of matcha marketed in Japan (mg/g)

No.	Theanine	Arg	Glu	Asp	Asn	Ser	Gln	GABA	Caffeine	EGCG	ECG	EGC	EC
1	22.33	2.96	3.01	3.57	0.52	0.71	0.61	0.14	33.31	63.08	8.58	21.65	4.22
2	19.48	4.25	3.09	4.56	1.13	1.10	0.65	0.14	36.28	66.06	9.30	17.74	3.81
3	15.81	2.87	2.72	3.96	0.82	1.10	0.67	0.20	39.95	74.80	11.07	21.63	4.60
4	38.77	18.19	7.58	8.47	3.36	1.84	1.34	0.22	44.43	57.20	13.81	9.68	3.44
5	31.53	10.92	5.72	7.25	2.44	1.07	0.92	0.23	39.32	57.14	12.49	12.73	3.81
6	12.29	2.07	3.47	3.47	0.79	0.74	0.63	0.25	35.24	75.15	11.34	26.33	5.14
7	23.34	7.14	5.48	6.25	1.32	0.97	0.75	0.15	38.73	64.06	10.30	12.73	2.50
8	25.53	7.81	5.19	6.21	1.37	1.00	0.80	0.15	37.17	63.00	10.46	13.33	2.93
9	14.79	1.81	2.93	3.87	0.56	0.74	0.77	0.25	35.47	74.65	12.30	25.76	5.95
10	31.09	10.92	5.92	5.98	1.21	0.92	1.55	0.27	33.66	60.99	10.35	12.96	3.19
11	2.48	0.36	0.48	0.65	0.09	0.13	0.11	0.05	5.95	13.15	2.20	4.09	0.87
12	22.63	4.52	4.63	4.71	0.86	0.67	0.62	0.17	36.11	67.91	11.01	10.45	2.63
13	21.03	6.76	3.88	5.01	1.24	1.06	1.42	0.13	43.85	78.38	12.76	15.20	3.51
14	27.60	15.44	7.63	7.40	4.14	1.46	1.04	0.14	39.87	55.63	12.20	12.35	3.84
15	25.48	8.23	3.48	6.04	1.75	1.04	0.95	0.22	39.61	67.67	12.25	12.64	3.38
16	27.99	12.15	7.80	6.95	2.66	1.20	0.98	0.24	39.84	60.56	12.64	12.70	3.82
17	20.39	4.69	5.22	4.57	0.80	0.76	0.76	0.25	36.00	70.90	11.50	15.25	3.36
18	28.99	12.50	7.52	6.95	2.90	1.25	1.08	0.25	39.95	59.34	12.44	14.86	4.26
19	31.64	14.53	8.11	6.91	2.60	1.21	1.21	0.32	37.43	55.76	12.21	7.80	2.66
20	21.73	7.97	5.67	5.77	1.91	0.98	1.02	0.44	37.68	61.66	11.12	11.19	2.93
21	34.50	15.34	8.34	7.15	3.86	1.45	1.09	0.29	39.07	54.71	12.55	9.79	3.19
22	10.62	1.25	2.83	2.14	0.44	0.68	1.17	0.15	24.28	63.45	11.65	39.05	10.46
23	7.25	0.31	3.19	1.39	0.12	0.46	0.43	0.20	15.18	52.70	10.00	42.95	10.28
24	10.76	0.78	2.39	1.71	0.47	0.55	1.03	0.19	17.64	56.17	10.21	41.10	10.75
25	29.31	12.88	4.80	6.98	1.74	1.06	2.13	0.19	41.98	66.03	13.18	12.49	4.01
26	17.53	3.89	3.69	3.66	0.78	0.89	0.95	0.25	32.90	59.90	9.21	20.42	4.33
27	34.29	15.04	8.32	7.14	3.81	1.45	1.09	0.29	37.80	52.83	12.03	9.47	3.05
28	22.19	7.08	5.19	4.74	1.27	1.04	2.17	0.51	36.41	60.30	10.46	11.33	2.87
29	11.22	2.14	3.41	3.50	0.82	0.83	0.65	0.20	34.63	71.83	12.47	27.09	6.22
30	33.64	17.97	10.91	8.50	3.36	2.09	4.63	0.23	44.56	64.08	13.30	6.42	2.06
31	32.05	14.43	7.36	7.39	2.59	1.47	3.59	0.26	38.74	59.20	12.73	9.61	2.98
32	28.81	14.86	8.96	7.16	3.90	1.49	1.77	0.30	40.85	59.42	12.99	14.42	4.01
33	21.82	5.50	4.17	5.37	1.01	1.02	1.05	0.28	37.51	67.73	9.95	12.25	2.68
34	37.13	17.05	10.06	8.20	2.38	1.85	3.03	0.18	43.15	61.97	13.55	7.37	2.28
35	33.52	15.77	8.56	7.39	3.10	1.44	2.09	0.22	39.26	58.84	13.35	8.06	2.76
36	11.34	2.18	3.27	3.46	0.93	0.79	0.75	0.20	35.61	74.77	13.24	28.82	6.56
37	16.48	4.43	3.99	2.34	1.38	1.08	1.16	0.39	37.48	60.10	10.33	23.01	5.46
38	21.02	6.10	5.00	4.14	0.98	0.82	1.90	0.30	36.27	54.86	12.30	21.64	6.39
39	22.51	9.03	5.34	3.06	1.97	1.10	0.96	0.27	39.45	61.49	11.08	15.88	3.90
40	15.77	4.20	3.80	3.97	1.32	1.04	1.13	0.37	37.74	59.97	10.36	23.06	5.56
41	22.57	6.88	5.37	4.58	1.05	0.84	1.68	0.35	36.31	54.14	12.87	21.45	6.44
42	10.16	2.11	3.04	2.62	0.58	0.75	0.70	0.29	28.12	63.52	10.95	37.85	7.98
43	8.72	1.56	2.76	2.41	0.53	0.76	0.52	0.29	27.42	65.18	10.83	40.53	8.08
44	19.54	5.61	4.94	4.66	1.56	1.05	1.29	0.21	37.88	61.84	11.22	17.53	4.35
45	14.90	2.78	3.22	3.89	1.33	1.07	0.96	0.34	36.13	64.11	10.49	23.03	4.86
46	12.20	2.03	2.94	3.04	0.65	0.78	0.97	0.28	35.22	66.86	11.62	33.08	7.27
47	44.65	10.74	5.86	7.95	4.01	1.74	3.20	0.23	37.19	48.44	9.96	8.40	2.50
48	21.76	8.21	5.00	5.86	2.03	0.93	1.37	0.34	40.78	63.61	10.89	13.06	3.06
49	24.36	8.50	4.99	3.04	1.31	0.94	1.41	0.19	39.93	69.18	13.03	13.53	3.53
50	16.38	3.14	3.38	3.21	0.44	0.67	1.05	0.17	36.01	74.69	13.02	21.55	4.70
51	10.17	3.51	3.46	3.92	1.38	0.77	0.74	0.33	40.31	76.39	13.19	21.77	5.01
52	7.87	1.41	2.64	2.80	0.59	0.57	0.51	0.14	40.35	86.76	16.09	28.76	6.48
53	13.13	5.64	3.63	4.03	1.75	0.80	0.99	0.42	42.19	67.04	11.01	16.02	3.79
54	22.46	2.17	4.39	2.45	0.33	0.54	0.89	0.22	43.18	84.31	12.86	39.20	7.24
55	14.24	3.49	3.63	4.08	0.59	0.71	0.83	0.19	38.86	84.47	15.87	26.15	5.60
56	19.54	5.47	5.05	4.82	1.22	0.91	0.69	0.22	38.55	63.66	10.66	13.84	3.00
57	28.35	12.14	9.27	7.85	2.24	1.42	1.00	0.22	39.86	59.04	12.07	7.28	2.10
58	23.12	11.66	7.14	6.85	2.61	1.09	0.97	0.20	40.47	58.16	12.84	13.49	3.96
59	17.57	9.61	6.65	7.02	2.98	1.21	0.64	0.16	44.65	61.68	12.82	18.28	4.87
60	17.68	7.20	4.59	6.05	3.30	1.42	0.59	0.16	46.08	65.87	12.58	19.85	4.97
61	27.57	12.90	8.34	6.77	1.98	1.47	1.32	0.12	50.16	72.69	14.31	6.77	0.87
62	28.22	13.98	7.90	6.49	2.33	1.10	1.93	0.15	38.55	56.51	13.92	10.21	2.60
63	18.55	7.20	4.83	6.16	1.64	1.02	0.56	0.19	40.02	61.11	10.02	15.75	2.92
64	11.18	6.42	4.71	5.43	2.23	0.97	0.47	0.20	37.95	57.39	9.37	20.07	3.66
65	12.84	5.30	4.76	4.91	3.34	1.21	0.27	0.21	37.42	51.68	11.21	30.12	7.30
66	17.44	7.17	5.97	6.59	1.71	1.22	0.53	0.08	40.02	67.07	11.59	15.77	2.42
67	12.46	4.74	4.70	4.58	1.43	0.90	0.42	0.09	35.21	60.91	9.41	20.78	3.29
68	18.24	7.35	4.79	5.71	2.69	1.21	0.49	0.20	41.14	60.17	10.73	16.92	3.60
69	12.90	4.28	3.57	4.77	1.55	1.04	0.44	0.15	37.20	63.71	10.05	22.84	3.88
70	24.82	11.47	7.10	6.56	3.16	1.31	1.36	0.21	39.37	56.54	12.34	13.33	3.29
71	25.23	14.24	4.31	5.64	4.80	2.87	2.97	0.18	37.09	47.69	7.36	14.27	3.09
72	40.62	15.36	10.57	12.16	3.74	2.15	1.38	0.29	37.03	66.40	10.47	12.52	2.20
73	18.23	8.67	6.41	6.54	2.49	1.09	0.53	0.11	40.43	56.19	11.95	17.20	4.16
74	9.44	2.31	3.05	4.65	0.82	0.69	0.53	0.12	36.95	71.31	10.43	23.32	4.24
75	19.66	9.27	5.63	6.40	3.29	1.39	0.49	0.19	41.09	56.15	11.30	16.90	3.96
76	8.89	1.20	1.81	1.45	0.17	0.46	1.07	0.12	32.38	87.46	16.37	44.43	10.19

Table s2. Components in matcha marketed in abroads (mg/g)

No.	Theanine	Arg	Glu	Asp	Asn	Ser	Gln	GABA	Caffeine	EGCG	ECG	EGC	EC
1	4.86	0.34	2.21	1.75	0.29	0.44	0.46	0.07	23.89	69.68	13.71	40.37	11.17
2	9.65	1.20	2.76	2.32	0.55	0.56	0.80	0.23	25.48	66.16	11.98	39.04	9.21
3	16.21	7.02	5.30	6.58	3.09	1.43	0.37	0.21	39.36	58.16	10.55	21.34	4.97
4	4.14	0.32	2.35	1.41	0.22	0.48	0.36	0.15	17.81	56.19	10.55	46.21	14.02
5	13.42	1.47	3.14	2.02	0.52	0.55	2.60	0.21	38.43	80.27	15.99	37.68	10.54
6	4.86	0.41	2.08	1.59	0.10	0.40	0.34	0.23	16.77	61.37	10.39	48.79	10.10
7	6.62	0.29	2.39	1.43	0.08	0.29	0.33	0.08	14.71	57.76	10.15	48.59	10.67
8	0.75	0.06	0.27	0.14	0.02	0.04	0.05	0.01	1.66	6.74	1.31	5.18	0.92
9	9.75	1.84	3.04	2.64	0.96	0.75	0.11	0.21	33.25	72.23	12.05	28.83	6.15
10	14.67	2.89	3.33	3.66	0.56	0.83	0.77	0.13	40.96	86.66	14.57	27.33	5.64
11	6.92	0.61	2.01	1.74	0.29	0.68	1.44	0.12	28.89	76.68	14.80	47.68	12.22
12	4.55	0.35	2.22	1.31	0.20	0.29	0.42	0.08	21.19	65.64	13.96	32.30	8.80
13	6.26	0.31	2.71	1.80	0.19	0.82	0.71	0.08	22.12	58.27	11.88	53.20	14.33
14	7.33	0.30	2.10	1.84	0.34	0.49	0.78	0.13	30.05	81.56	16.07	39.29	10.36
15	13.98	2.68	3.16	3.59	0.93	0.77	0.86	0.36	37.89	76.14	11.66	28.14	5.63
16	27.09	9.75	4.13	4.19	2.21	0.89	2.14	0.39	38.88	78.07	13.33	26.45	5.78
17	5.13	0.25	2.03	0.61	0.21	0.29	0.93	0.11	21.38	73.76	15.85	40.04	10.74
18	8.81	1.65	2.53	2.59	0.87	0.60	0.82	0.24	34.91	74.59	14.24	34.17	8.28
19	4.32	0.59	1.60	0.95	0.31	0.23	0.32	0.07	16.94	65.01	14.14	24.74	7.50
20	10.50	1.71	2.60	2.60	0.76	0.60	1.32	0.13	29.44	65.56	11.48	36.39	8.55
21	0.32	0.03	0.16	0.08	0.01	0.03	0.03	0.01	0.85	3.02	0.63	2.25	0.18
22	0.82	0.12	0.33	0.24	0.07	0.06	0.08	0.03	3.58	9.51	1.91	5.47	1.04
23	7.79	2.84	2.35	2.01	1.46	0.96	0.42	0.13	35.76	58.37	9.96	27.78	6.59
24	17.68	2.18	4.47	3.27	0.76	0.61	0.89	0.09	34.88	88.23	21.01	28.28	8.33
25	14.63	2.45	2.65	2.60	3.59	1.50	0.86	0.27	37.49	61.24	12.89	35.72	11.03
26	4.26	0.59	1.62	1.47	0.34	0.41	0.67	0.07	28.96	82.94	16.26	47.65	13.00
27	7.04	0.74	2.41	1.75	0.38	0.32	0.70	0.07	30.47	79.56	17.70	33.05	9.34
28	4.94	0.43	2.14	1.65	0.33	0.41	0.57	0.06	22.91	70.86	14.00	39.05	10.32
29	4.82	0.44	2.08	1.26	0.17	0.32	0.53	0.07	19.18	62.29	14.74	42.23	12.68
30	12.74	4.83	3.11	3.37	2.19	0.85	1.07	0.20	31.49	58.79	11.46	12.74	3.72
31	16.30	5.32	3.78	3.53	1.92	0.84	1.57	0.46	39.00	73.66	11.74	26.76	5.87
32	17.79	2.68	4.08	2.98	1.00	0.60	1.17	0.08	33.01	85.83	19.33	30.60	8.59
33	4.62	0.44	2.31	1.15	0.13	0.24	0.75	0.08	21.89	51.50	11.15	28.30	9.54
34	17.05	8.66	5.34	4.19	1.75	0.79	1.84	0.27	34.85	59.91	11.15	11.08	2.69
35	15.55	1.74	2.41	2.02	0.86	0.68	1.39	0.12	37.61	80.44	13.56	30.66	7.62
36	3.84	0.08	1.85	1.01	0.10	0.28	0.24	0.05	23.65	83.01	15.77	35.28	9.21
37	15.26	1.81	3.25	3.32	0.70	0.75	0.79	0.18	38.16	86.39	14.21	28.54	5.77
38	8.73	1.50	2.44	2.50	0.58	0.51	0.61	0.23	34.23	76.61	13.62	35.01	8.07
39	7.67	1.22	2.82	2.88	1.62	0.94	0.59	0.18	36.70	79.24	13.77	33.02	7.14
40	11.91	1.66	2.84	2.19	0.47	0.52	1.77	0.16	36.36	80.87	16.19	43.51	11.74
41	4.94	0.97	1.40	1.52	0.37	0.34	0.28	0.07	16.95	37.38	6.43	15.47	3.51
42	7.21	1.21	1.24	1.13	0.16	0.30	1.17	0.05	13.27	28.50	5.10	12.32	3.08
43	17.20	4.29	3.80	4.22	1.44	0.99	1.01	0.43	38.44	65.43	10.00	27.13	5.38
44	12.19	3.14	3.39	3.90	1.03	0.79	0.94	0.16	34.80	70.38	12.13	28.08	6.43
45	4.07	0.19	1.92	1.10	0.10	0.25	0.34	0.08	21.00	66.75	14.58	39.09	11.31
46	5.25	0.39	2.27	1.51	0.21	0.29	0.28	0.09	23.32	60.86	14.71	46.88	15.40
47	2.69	0.14	1.35	0.89	0.15	0.21	0.21	0.02	22.46	53.84	18.56	27.34	13.70
48	4.71	0.21	1.97	1.04	0.06	0.20	0.32	0.04	17.36	72.94	12.14	38.91	9.06
49	16.04	2.92	2.73	4.03	0.86	1.13	0.67	0.19	39.84	75.27	11.01	22.33	5.12
50	3.81	0.15	1.81	0.99	0.12	0.36	0.32	0.22	19.11	67.59	11.22	54.22	11.37
51	3.12	0.12	1.45	0.80	0.10	0.29	0.25	0.17	4.46	8.12	1.20	1.86	0.39
52	20.56	4.80	4.50	4.65	1.15	0.86	0.88	0.34	32.81	58.32	9.11	15.70	3.83
53	15.13	2.29	3.30	3.58	0.84	0.83	0.91	0.22	36.17	73.61	12.53	26.16	6.31
54	13.23	2.24	3.09	2.79	0.66	0.70	0.76	0.25	28.75	64.76	11.05	33.54	7.89
55	4.73	0.36	2.02	1.01	0.18	0.27	0.38	0.07	14.52	49.84	9.52	43.16	10.70
56	5.61	0.31	2.24	1.19	0.06	0.30	0.71	0.08	16.13	56.14	10.51	48.69	11.85
57	4.32	0.15	1.81	1.09	0.11	0.29	0.59	0.06	23.03	87.15	15.65	32.78	8.37
58	6.66	0.42	2.40	1.25	0.08	0.29	0.40	0.05	17.50	73.47	13.38	38.17	9.36
59	6.13	0.38	2.07	1.84	0.34	0.40	0.88	0.10	23.27	70.58	12.67	40.66	10.17
60	4.01	0.88	1.61	1.08	0.40	0.33	0.30	0.06	24.43	69.11	22.71	33.61	14.08
61	13.71	2.97	2.63	1.78	0.20	0.80	2.81	0.20	28.15	84.29	27.96	32.75	13.58
62	8.45	0.89	2.86	2.07	0.12	0.26	0.57	0.04	25.80	76.14	9.86	28.89	5.67
63	3.53	0.17	2.10	1.44	0.16	0.38	0.51	0.06	24.41	67.12	14.01	48.93	12.66
64	3.66	0.26	1.50	1.02	0.09	0.19	0.51	0.14	14.25	62.38	10.69	50.17	11.22
65	7.96	0.60	2.27	1.48	0.28	0.42	1.44	0.17	25.03	74.06	14.98	39.68	10.26
66	5.45	0.67	2.15	1.32	0.30	0.47	0.76	0.13	25.06	68.16	13.60	45.27	10.81
67	7.20	0.96	2.09	1.35	0.40	0.53	0.76	0.09	27.07	65.89	13.06	44.42	11.37
